# Lnc-CNNM3-DT as a protective factor in cervical cancer: regulation of LIAS expression and intracellular copper levels

**DOI:** 10.3389/fonc.2025.1571788

**Published:** 2025-04-08

**Authors:** Ying Yang, Xuehong Zhu, Dan Sun, Jiangtao Fan

**Affiliations:** ^1^ Department of Gynecology and Obstetrics, The First Affiliated Hospital of Guangxi Medical University, Nanning, Guangxi, China; ^2^ Department of Gynecology, Yulin First People’s Hospital (The Sixth Affiliated Hospital of Guangxi Medical University), Yulin, Guangxi, China; ^3^ Department of Reproductive Medicine, Reproductive Hospital of Guangxi Zhuang Autonomous Region, Nanning, Guangxi, China

**Keywords:** cervical cancer, cuproptosis, lncRNA, lipoic acid synthase, prognostic marker

## Abstract

**Background:**

Cervical cancer (CC) is the fourth leading cause of cancer-related death in women globally.While early screening has reduced mortality, tumor metastasis remains a significant concern, particularly in developing countries. Recent studies have identified cuproptosis, a copper-dependent cell death mechanism, as a potential factor in tumor progression. Long non-coding RNAs (lncRNAs) are key regulators of tumor progression. This study investigates the role of cuproptosis-related lncRNA (CRL) CNNM3-DT in CC, focusing on its impact on LIAS expression, intracellular copper levels, and tumor progression.

**Methods:**

We analyzed the expression of lnc-CNNM3-DT and LIAS in clinical samples and CC cell lines using Real-time Polymerase Chain Reaction (RT-qPCR), Western blot, and immunohistochemistry (IHC). Functional assays, including CCK-8, wound healing, transwell invasion, and flow cytometry, were used to evaluate the effects of lnc-CNNM3-DT overexpression on cell proliferation, migration, invasion, and apoptosis. Intracellular copper ion levels were measured, and correlations between lnc-CNNM3-DT, LIAS, and clinicopathological features were analyzed.

**Results:**

Lnc-CNNM3-DT expression was significantly higher in paracancerous tissues and normal cervical epithelial cells compared to tumor tissues and CC cell lines. Overexpression of lnc-CNNM3-DT suppressed proliferation, migration, and invasion of HeLa and SiHa cells while enhancing apoptosis. Additionally, lnc-CNNM3-DT overexpression downregulated LIAS expression and decreased intracellular copper ion levels. Correlation analysis indicated that lnc-CNNM3-DT expression was negatively associated with tumor diameter and depth of invasion, while LIAS expression showed no significant correlation with clinicopathological features.

**Conclusion:**

Our findings suggest that lnc-CNNM3-DT functions as a protective factor in CC by inhibiting tumor progression through downregulation of LIAS expression and reduction of intracellular copper levels. These results highlight lnc-CNNM3-DT as a potential biomarker and therapeutic target in CC.

## 1 Introduction

CC has been the fourth leading reason for cancer-related mortality in women globally. Though current early screening has reduced mortality in patients with CC, tumor metastasis continues to occur and its incidence remains high in many developing countries (1). Almost all CC are attributable to infection with high-risk HPV (2); however, the presence of the virus alone is not sufficient to promote tumor progression. 85-90% of high-risk HPV infections spontaneously clear, and only 10-15% persist, promoting the progression of cervical intraepithelial neoplasia to invasive CC, suggesting that other co-factors are also required for CC progression (3). Cu is an essential mineral and co-factor for eukaryotes (4, 5), and Cu imbalance (excess or deficiency) is associated with many diseases, including tumorigenesis and increased aggressiveness of cancer (4). Cu can enhance cell proliferation by activating the RAS/RAF/MEK/ERK signaling pathway and to stimulating angiogenic factors, thereby facilitating tumor progression and metastasis (6, 7).

Tsvetkov et al. (8) recently identified cupropsis as a new Cu-dependent regulatory mechanism potentially related to tumorigenesis. Cupropsis relies on mitochondrial respiration, where Cu directly interacts with the lipid component of the tricarboxylic acid cycle, due to the aggregation of lipoacylated proteins and loss of iron-sulfur cluster proteins, resulting in proteotoxic stress and cell death. Studies showed that the metabolism of tumor cells, is significantly correlated with oxidative phosphorylation in mitochondrial respiration, as shown by the reversal of the Warburg effect (9, 10). Abnormally elevated ROS levels in tumor cells down-regulate cellular antioxidant enzyme systems and promote tumor proliferation, growth, and distant metastasis (11–13). Most ROS originate from intracellular REDOX reactions, in which mitochondria play a major role (14). It is also consistent with the correlation between cupropsis and mitochondrial metabolism.

Tsvetkov et al. also found seven genes related to cupropsis including LIAS, an upstream regulator of cupropsis, and knockout of LIAS resulted in cupropsis resistance in cells (15). LIAS is a family member of biotin-lipoic acid synthetases and catalyzes the last step of lipoic acid synthesis (16). GO and KEGG enrichment analysis showed that LIAS-related genes were mainly aggregated in some cancer-related pathways, especially mitochondrial matrix and nucleotide excision repair (17). LIAS can regulate mitochondrial energy metabolism and oxidative stress, and affect the proliferation, migration, angiogenesis, and immune block of cancer cells (16). Some studies showed that LIAS expression is upregulated in lung adenocarcinoma, hepatocellular carcinoma, cholangiocarcinoma, and other tumors. It was down-regulated in endometrial carcinoma, thyroid carcinoma, breast cancer, renal papillary cell carcinoma, prostate adenocarcinoma, rectal adenocarcinoma, and so on. There was no significant change in LIAS expression in other cancers such as cervical squamous cell carcinoma, endocervical adenocarcinoma, uterine carcinosarcoma, and so on (17). LIAS may have a potentially critical role in regulating biological functions in cancer.

LncRNA is a transcript exceeding 200 nucleotides that do not code for proteins and are linked to various human cancers, including CC (18). IncRNA can regulate chromatin remodeling, histone modification, or DNA methylation to activate or silence genes through various mechanisms, such as epigenetics, transcriptional regulation (such as transcriptional interference and transcriptional activation), and post-transcriptional regulation (19). They are crucial in regulating the cell cycle, differentiation, and tumor development (20). Some lncRNA molecules have been novel prognostic indicators to evaluate the survival of cancer patients (21, 22). However, the role of CRLs in the pathogenesis of CC remains uncertain. This study investigates the role of CRL CNNM3-DT in CC mechanisms, enhancing the understanding of prognostic biomarkers and treatment strategies for CC patients.

Liu et al. (23) utilized the TCGA database and Genotype-Tissue Expression Engineering (https://gtexportal.org/home/) to identify seven factors significantly associated with the prognosis of CC, constructing a prognostic model. The prognostic significance of this signature was confirmed by CC tissues. The CRL CNNM3-DT served as a protective factor. Combined with the consequences of the previous bioinformatics analysis of our research group and Liu’s research, lnc-CNNM3-DT was selected for subsequent experiments.

## 2 Materials and methods

### 2.1 Clinical samples

Twenty-four cases of CC tumor and paracancerous tissues were collected from the Guangxi Medical University First Affiliated Hospital, and the clinicopathological information of the patients was recorded. Inclusion criteria: patients diagnosed with primary CC who are undergoing surgical treatment for the first time, have not received any radiotherapy or chemotherapy, and have no other internal or external complications. The collection sites include CC tumor tissue and paracancerous tissue located more than 3 cm away from the tumor tissue. Exclusion criteria: patients with other malignant tumors in addition to the confirmed diagnosis of primary CC; non-first-line treatment, meaning those who have received chemotherapy, radiotherapy, or other therapies before surgical intervention; patients with other severe systemic diseases. All the subjects signed the informed consent, and the ethics committee (Ethics Review Approval number: 2022-K11-01) reviewed and approved the implementation.

### 2.2 Cell Culture

HeLa, SiHa (presented by the Jiangtao Fan research group on October 10, 2022), and HUCEC cells (purchased from Nanjing Wanmuchun Biotechnology Co., LTD., Nanjing, China, catalog no. C1481, product format: a T25 flask). Three types of cells were identified by Short Tandem Repeat and were free of mycoplasma infection. They were cultured in an environment of 37°C, 5% carbon dioxide, and 99% humidity. The culture medium consisted of Gibco DMEM high glucose medium, containing 10% fetal bovine serum (FBS) and 1% penicillin-streptomycin solution. The medium was refreshed every 24 to 48 hours. Subsequent experiments were carried out when the cells were in a good growth state.

### 2.3 RT-qPCR

Obtaining total RNA from tissues and cells using the NcmSpin Cell/Tissue Total RNA Kit (New Saimei Biotechnology Co., LTD., Suzhou, China).RNA was reverse transcribed using a Thermo Scientific Hyclone reverse transcription kit (Utah, USA).RT-qPCR proceeded with SYBR Green PCR Master Mix (Applied Biosystems, USA), using CNNM3-DT as the primer and GAPDH as the internal control. Normalization was performed by the 2^−ΔΔCT^ method.The primer sequences in RT-qPCR were as follows:

CNNM3-DT: F 5’-CCTCAGCACACTCAATCG-3’,R 5’-CCTTTTCGTCCACACCTA-3’;
GAPDH: F 5’-AATCAAGTGGGGCGATGCTG-3’,R 5’-GCAAATGAGCCCCAGCCTTC-3’.

### 2.4 The western blot assay

Protein extraction from tissues and cells was performed with a total protein extraction kit (Beijing Adlai Biotechnology Co., LTD., Beijing, China). SDS-PAGE protein loading buffer was added and boiled for 10 minutes. Electrophoresis was carried out at 160V for 50min, followed by membrane transfer at 300 mA for 35min. Membranes were blocked for 2 hours using 5% skim milk powder, stepped by an overnight incubation with primary antibodies at 4°C. The concentration of the primary antibody was LIAS (1:2000, 11577-1-AP, proteintech).α-Tubulin (1:5000, 11224-1-AP, proteintech).Then, incubating with fluorescent secondary antibodies at room temperature in the dark for 1h was conducted. The PVDF membrane was scanned with an Odyssey infrared fluorescence imaging scanner and the images were saved. Image J software was used to analyze the gray value of the bands. Relative expression of LIAS = IOD of LIAS/IOD value of α-Tubulin band.

### 2.5 IHC

Clinical tissue samples, comprising both tumor and paracancerous tissues, were fixed in 4% paraformaldehyde and then embedded in paraffin. IHC staining of LIAS (1:100, 11577-1-AP, proteintech) and α-Tubulin (1:100, 11224-1-AP, proteintech) was performed on sections. DAB was added dripping, counterstained with Mayor’s hematoxylin, and sealed with neutral gum. Images were taken under a light microscope with a magnification of 10× and 40×. LIAS were stained as brown areas.

### 2.6 Correlation analysis

SPSS 22.0 was employed for correlation analysis. For quantitative variables, Pearson correlation analysis was utilized, while Spearman correlation analysis was applied to ordinal variables. The aim was to analyze the correlations among lnc-CNNM3-DT, LIAS, and the clinicopathological features of CC. A sample correlation coefficient of r>0 revealed a positive correlation, and r < 0 revealed a negative one. Specifically, 0≤/r/<0.3 meant no correlation; 0.3≤/r/<0.5 signified a slight correlation; 0.5≤/r/<0.8 represented a moderate correlation; and 0.8≤/r/<1 indicated a significant correlation. *P*< 0.05 represents an overall correlation between the two variables.

### 2.7 Cell transfection

The stable cell lines of HeLa and SiHa overexpressing lnc-CNNM3-DT were established by transfection of the constructed overexpression lentivirus (bought from Suzhou Jima Gene Co., LTD., Suzhou, China). The gene name of the overexpressed lentivirus was CNNM3-DT homo, the vector type was LV5(EF-1a/GFP&Puro), the sequence was NR_149141.1, and the titer was 1x10 ^8^. The control virus LV5-NC was empty, with no inserted sequence, and the titer was 1x10 ^8^. The cells in the logarithmic growth phase were seeded at 1×10^5^ cells/well in a 24-well plate and incubated for 24 hours in a cell incubator at 37°C. The corresponding volume of the virus was added according to the virus volume =(MOI× number of cells)/virus titer, and the Polybrene concentration was adjusted to 5ug/ml. After 24 hours of infection, the culture was replaced with a complete medium and continued. The efficiency of infection was observed by fluorescence microscopy at 72 hours after infection, and the cells were replaced with a complete medium containing 2μg/mL puromycin for further screening and expansion, while the cells were collected for subsequent RT-qPCR identification. The blank (HeLa or SiHa) and empty vector group (lnc-NC group) were set as controls.

### 2.8 Determination of intracellular copper levels

According to the manufacturer’s instructions (Product item number: E-BC-K775-M), the concentration of copper level in cells was determined using a cell copper colorimetric test kit (Elabscience, Wuhan, China). Collect the cells and add 0.2 mL of lysis buffer for approximately every 2×10^6^ cells, mix well, and place on ice to lyse for 10 minutes. Then centrifuge at 4°C, 12000×g for 10 minutes, and collect the supernatant for measurement, reserving a portion of the supernatant for protein concentration determination using the BCA method (measure the OD value of each well at 562 nm). Take 100 µL of eight different concentrations of standards and 100 µL of the samples to be tested, and add 50 µL of the color reagent working solution to each well. Cover with a membrane and incubate at 37°C for 5 minutes, then measure the OD value of each well at 580 nm using a microplate reader. Calculation of results: Standard curve fitting: y=ax+b, the formula for calculating the concentration of copper ions in cells: Copper level (μmol/gprot) = (δA580-b) ÷ a × f ÷ C_pr_. y: OD value of the standard well - OD value when the standard concentration is 0; x: concentration of the standard; a: slope of the standard curve; b: intercept of the standard curve; δA580: OD value of the sample measurement - OD value when the standard concentration is 0; f: dilution factor of the sample before adding to the detection system; C_pr_: protein concentration of the sample before adding to the detection system (gprot/L).

### 2.9 The CCK-8 assay

Three groups: blank (HeLa or SiHa), lnc-NC, and lnc-CNNM3-DT overexpression group, with a cell density of 4×10^4^ cells/ml, were inoculated in 96-well plates according to 100uL per well. Add PBS around each 96-well plate to prevent the evaporation of liquid in the experimental wells from affecting the experimental results. Additionally, set up three control wells for each 96-well plate (100 µl complete culture medium). Each group is vaccinated with three wells and each set of experiments is repeated three times on the same culture plate. At 8, 24, 48, and 72 hours of culture, 10 µL of CCK-8 solution was added, followed by a 2-hour incubation at 37°C. The OD value of each well was assessed at a wavelength of 450 nm by a microplate reader. Summarize the OD values of each group, subtract the OD values of the control wells, calculate their average to obtain the final result, and then plot the cell proliferation curve.

### 2.10 The flow cytometry assay

Take 1 million cells into 1 mL of PBS, mix well, centrifuge at 1200 rpm for 5 minutes, and discard the supernatant. Add 100 µL of 1× Binding Buffer to each tube, resuspend, and add 5 µL of Annexin V-PE and 5 µL of 7-AAD, obtained from BD Biosciences (San Jose, CA, USA). Gently shake the solution and incubate in the dark at room temperature (25°C) for 15 minutes. Then, add 300 µL of 1× Binding Buffer and use a Beckman Coulter cytology S flow cytometer to detect cell apoptosis within 1 hour.

### 2.11 The wound healing assay

Cells were taken and inoculated at a density of 5×10^5^ cells/well in a 6-well plate. After continuing the culture for 24 hours, the cells covered the bottom of the wells. A sterile pipette tip was used to scrape the cell layer in a straight line against a ruler. The cells were washed three times with a basic culture medium without FBS to remove detached cells and debris. The remaining cells were cultured in DMEM high glucose medium containing only 2% FBS, placed in a 37°C, 5% CO2 incubator for further culture. The edges of the spontaneously migrating cells were observed, and photographs were taken at 0, 24, and 48 hours using an optical microscope (Olympus, Japan). The area of the scratch is calculated using Image J. The percentage of wound healing (%) = (initial scratch area - scratch area at a certain time point)/initial scratch area × 100%.

### 2.12 The cell invasion assay

Before inoculation, the upper chamber was coated with Matrigel at a dilution ratio of 1:8 with a serum-free DMEM medium. Cells were resuspended in a medium with 2% FBS and placed in the upper chamber, while the lower chamber received a medium with 20% FBS. Cells were cultured for 48 hours, fixed with methanol for 20 minutes, stained with crystal violet for 15 minutes, then photographed and counted using Image J.

### 2.13 The analysis of statistics

Experiments were repeated at least three times and data were expressed as mean ± SEM. SPSS 22.0 and GraphPad Prism version 10.0 were performed for statistical analysis. The Student’s t-test was employed for comparing two experimental groups, while one-way ANOVA was utilized for comparing multiple groups. *P* > 0.05 indicates no statistical significance (ns), while statistical significance is denoted by *p < 0.05, **p < 0.01, ***p < 0.001, and ****p < 0.0001.

## 3 Results

### 3.1 Lnc-CNNM3-DT was highly expressed in paracancerous tissues and HUCEC

**Figure 1:**
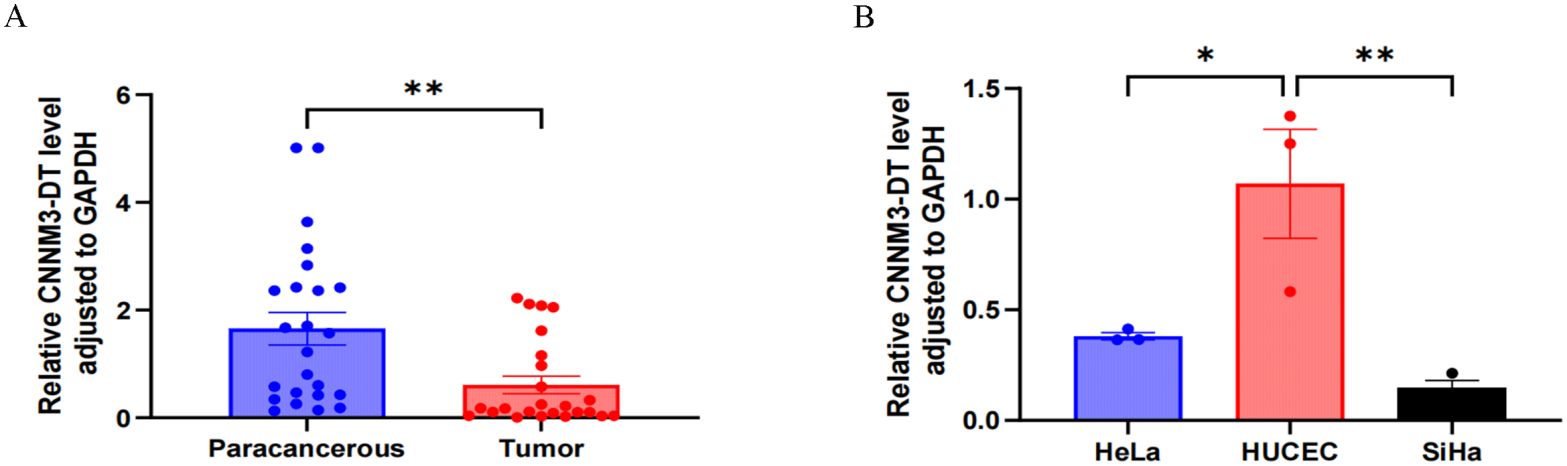
RT-qPCR was used to analyze the expression of lnc-CNNM3-DT in tumor tissues, paracancerous tissues, and three cell lines. RT-qPCR analysis showed that lnc-CNNM3-DT was highly expressed in paracancerous tissues **(A)** and HUCEC **(B)** (*p<0.05,** p<0.01).

The expression level of lnc-CNNM3-DT in 24 pairs of clinical tissue samples was examined by RT-qPCR. Paracancerous tissues showed a higher expression than tumor tissues, *p*<0.01 (**Figure 1A**). Analysis of lnc-CNNM3-DT expression showed elevated levels in the normal cervical epithelial cell line HUCEC compared to the CC cell lines HeLa (*p*<0.05) and SiHa (*p*<0.01, **Figure 1B**).

### 3.2 LIAS expression was elevated in tumor tissues, including HeLa and SiHa

**Figure 2:**
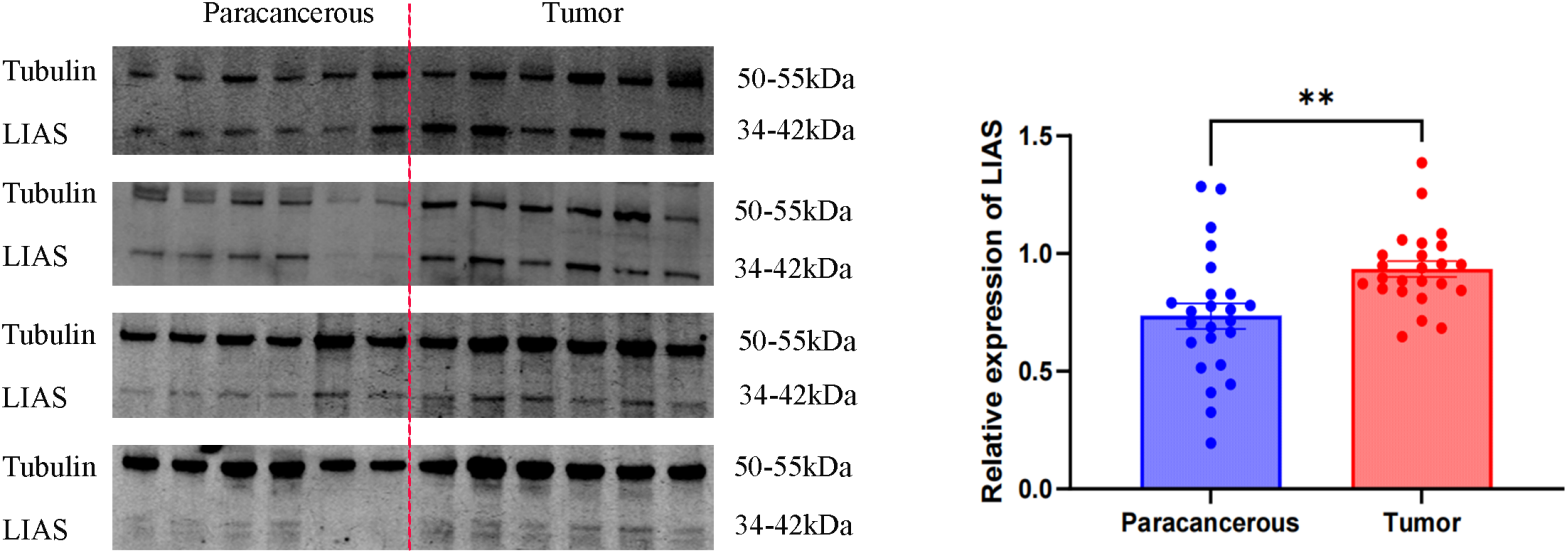
WB results of LIAS in 24 pairs of clinical tissue samples. WB experiments indicated LIAS was highly expressed in CC (**p < 0.01).

**Figure 3:**
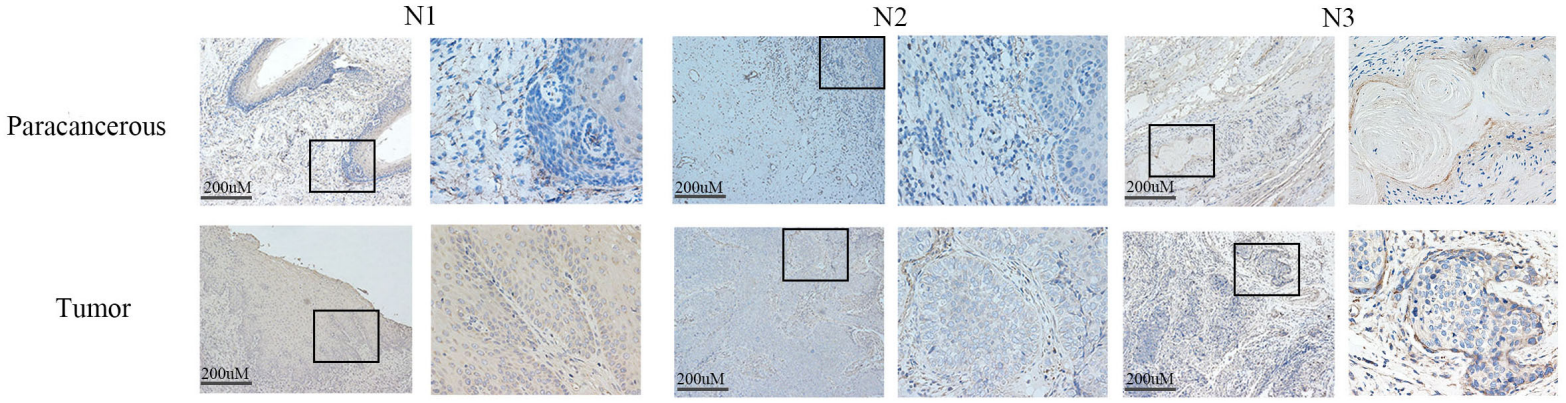
The expression of LIAS in cancer tissues and paracancerous tissues was demonstrated by IHC. IHC experiments showed that LIAS was highly expressed in tumor tissues. LIAS are strained as brown areas.

**Figure 4:**
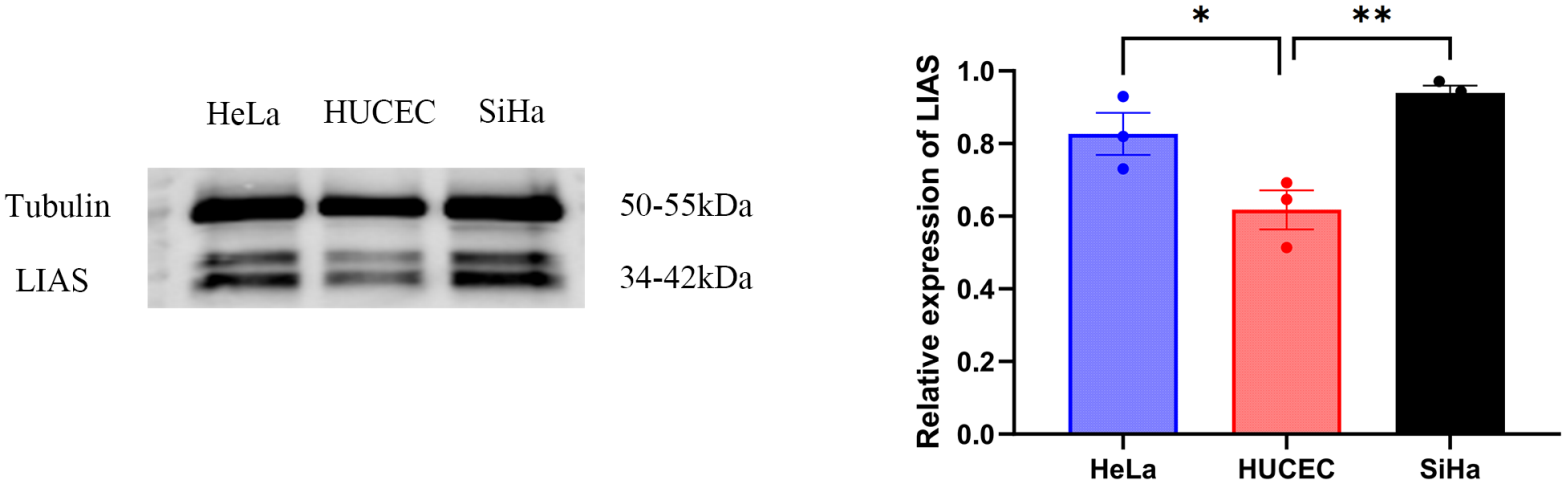
WB results of LIAS in three cell lines. WP experiments indicated LIAS was highly expressed in HeLa and SiHa (*p < 0.05, **p < 0.01).

Western blot analysis indicated elevated LIAS expression in tumor tissues, *p*<0.01 (**Figure 2**), and the same as the IHC (**Figure 3**). The results of WB experiments showed that LIAS was highly expressed in HeLa (*p*<0.05) and SiHa (*p*<0.01, **Figure 4**).

### 3.3 The expression of lnc-CNNM3-DT is negatively correlated with the tumor diameter and invasion depth of CC patients

Next, We analyzed the correlation between CC with LIAS and lnc-CNNM3-DT. **Table 1** presents the clinicopathological data of 24 patients diagnosed with CC. Correlation analyses using Pearson and Spearman methods indicated a negative association between lnc-CNNM3-DT expression and both tumor diameter and invasion depth (**Table 2**), while LIAS expression showed no correlation with the clinicopathological features of CC (**Table 3**).

**Table 1:** Clinicopathological characteristics of 24 patients.

No	Age (year)	FIGO Stage	Diameter of tumor (cm)	Depth of infiltration (cm)	Lymph node metastasis (number)	Vascular Metastasis	CA125 (U/ml)	SCC (ng/mL)	Relative expression of Lnc-CNNM3-DT	Relative expression of LIAS
1	50	IB2	2.5	1	0	none	11.3	0.9	2.0813819	0.9399773
2	59	IB1	1.2	0.4	0	yes	6.2	0.5	1.6179963	1.3859374
3	46	IB2	3.2	1.7	0	yes	31.4	1.4	0.1720470	0.9934378
4	55	IIA	3.1	1.5	0	yes	12.8	3.1	0.9698779	1.0440663
5	46	IB1	1	0.6	0	none	22.9	0.4	2.2230520	0.9531000
6	41	IB3	10	2.5	1	yes	21.2	18.4	0.0261788	0.8395574
7	58	IIB	3.6	1.7	0	none	16.4	5.7	0.3255328	1.0579088
8	58	IB2	3.8	1.7	0	yes	15.3	6.9	0.1072123	0.8952801
9	36	IB1	1	0	0	none	7.7	0.6	0.0095312	0.8502677
10	42	IB2	3.3	0.9	0	none	12.8	1.7	0.2176188	0.8839174
11	55	IB1	2	0.6	0	none	5.8	1	0.1009380	0.7149256
12	58	IB2	3.8	1.7	0	yes	15.3	6.9	0.0230707	0.8437257
13	64	IVA	3.5	1.6	0	none	14.8	2	0.0396796	0.9913209
14	55	IB2	2.5	0.8	0	none	10.1	1.8	0.0378615	0.9551436
15	57	IIA1	4	0.6	0	none	21.5	32.5	1.1555195	0.6465927
16	39	IIIC1p	5	0.8	1	yes	31.5	4.9	0.1028924	1.0849199
17	41	IB3	5.5	1.5	0	none	13.6	0.9	0.1141926	0.6832302
18	37	IIIC1p	7	2.2	4	yes	43.2	30.3	0.1761498	0.8714962
19	55	IB3	7.4	0.7	0	none	8.8	8	0.0341779	1.2550856
20	57	IIA1	3.3	0.7	0	none	7.1	0.6	2.0532012	0.8719353
21	59	IB2	1.7	1.2	0	none	16.3	0.6	0.2461384	1.0333384
22	71	IIIC1p	4.3	1.5	1	yes	31.5	17.4	0.5790962	0.9479778
23	49	IIA1	3.7	1.3	0	none	59.8	7.3	2.1109245	0.8825425
24	70	IIIC	4.5	2.3	1	yes	105	3.2	0.0853701	0.8094360

**Table 2:** Relationship between the expression level of lnc-CNNM3-DT with the clinicopathological features of CC patients.

Variaty	Pearson/Spearman	Sig.
Age (year)	0.053	0.806
FIGO stage	0.161	0.452
Diameter of tumor (cm)	-0.368	0.077
Depth of infiltration (cm)	-0.346	0.098
Lymph node metastasis (number)	-0.212	0.320
Vascular Metastasis	-0.171	0.425
CA125 (U/ml)	-0.015	0.945
SCC (ng/mL)	-0.084	0.696

**Table 3:** Relationship between the expression level of LIAS with the clinicopathological features of CC patients.

Variaty	Pearson/Spearman	Sig.
Age (year)	0.156	0.466
FIGO stage	0.031	0.884
Diameter of tumor (cm)	-0.119	0.579
Depth of infiltration (cm)	-0.194	0.365
Lymph node metastasis (number)	-0.095	0.660
Vascular Metastasis	0.098	0.650
CA125 (U/ml)	-0.201	0.346
SCC (ng/mL)	-0.298	0.157

### 3.4 Overexpression of lnc-CNNM3-DT suppressed HeLa and SiHa cell proliferation, migration, and invasion while enhancing apoptosis

**Figure 5:**
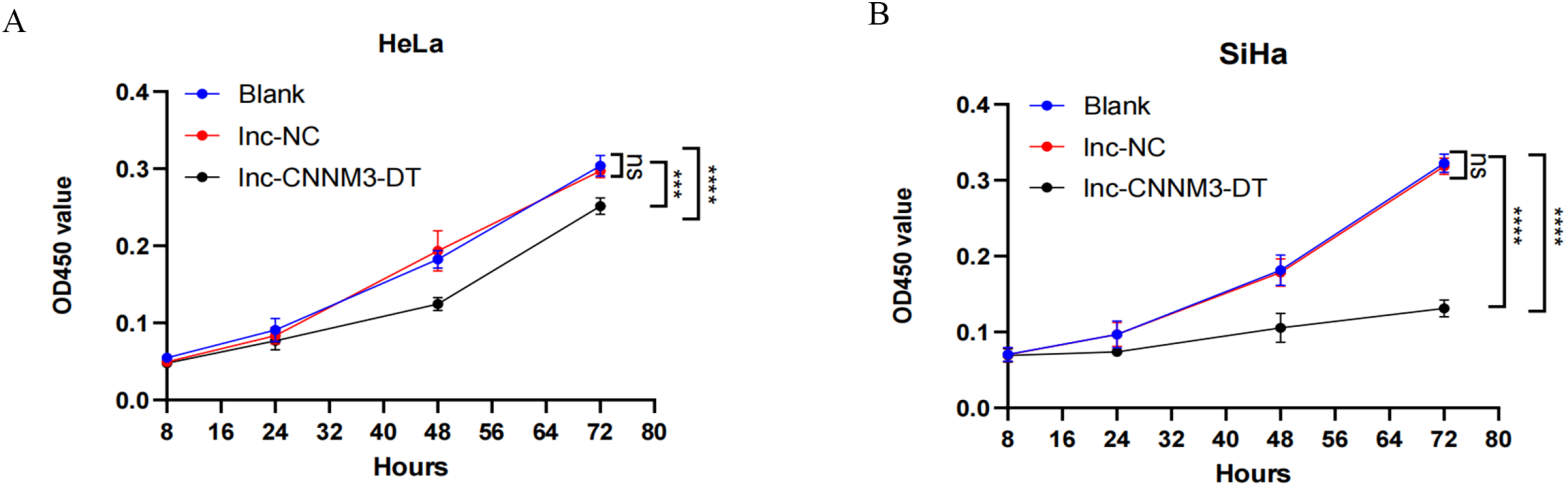
CCK8 assay showed the proliferation of HeLa and SiHa cell after overexpression of lnc-CNNM3-DT. The CCK-8 assay demonstrated that lnc-CNNM3-DT overexpression suppressed the proliferation of HeLa **(A)** and SiHa **(B)** (ns, p> 0.05, ***p< 0.001, ****p< 0.0001).

**Figure 6:**
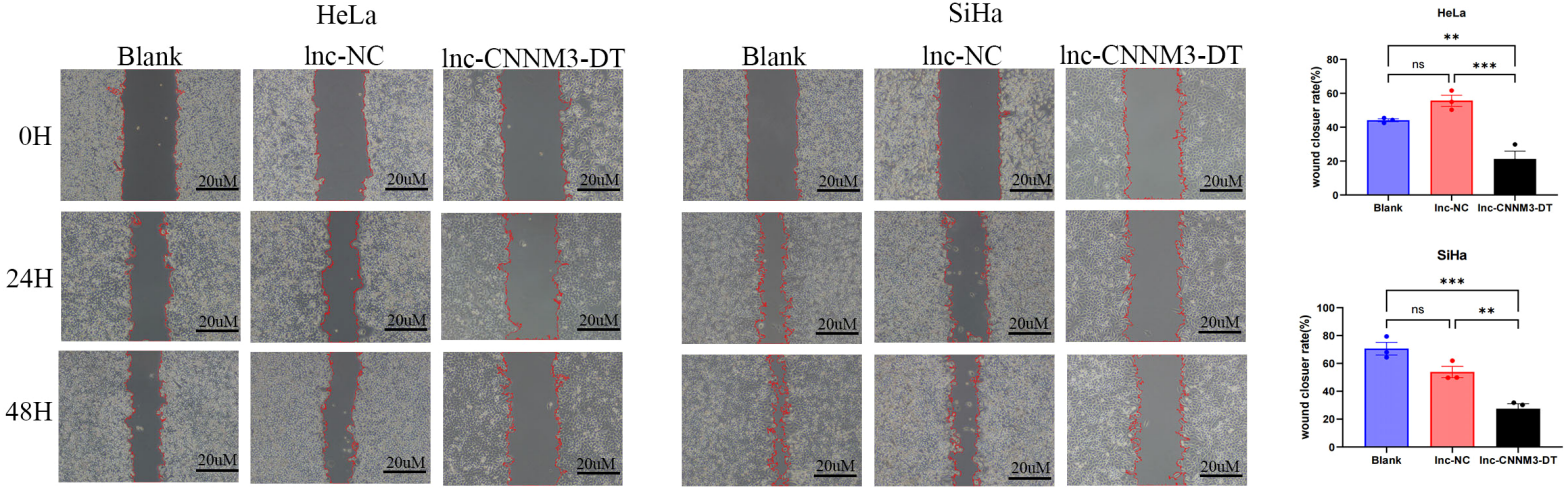
The wound healing assay demonstrated the migration of HeLa and SiHa cells after overexpression of lnc-CNNM3-DT. The wound healing assay revealed that overexpression of lnc-CNNM3-DT inhibited the migration of HeLa and SiHa (ns, p> 0.05, **p< 0.01, ***p <0.001).

**Figure 7:**
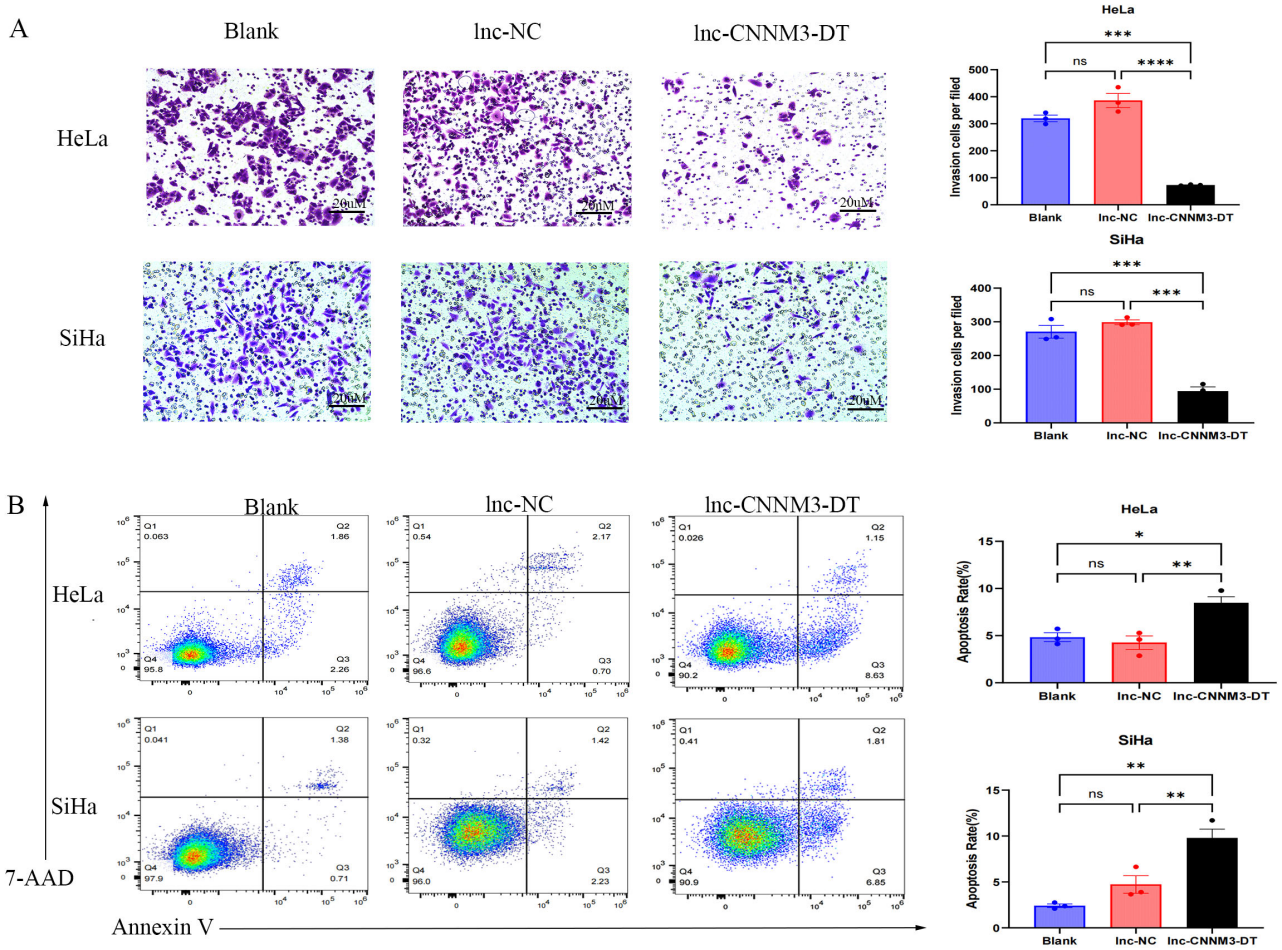
Matrigel Transwell invasion assay and Flow cytometry analysis demonstradted the invatsion and apoptosis of HeLa and SiHa overexpression of lnc-CNNM3-DT. Matrigel Transwell invasion assay revealed that overexpression lnc-CNNM3-DT inhibited the invasion of HeLa and SiHa **(A)**. Flow cytometry analysis demonstrated that Overexpression of lnc-CNNM3-DT. Matrigel transwell invation assay revlead that overexpression lnc-CNNM3-DT promoted the apoptosis of HeLa and SiHa **(B)** (ns, p > 0.05, *p <0.05, **p <0.01, ***p < 0.001, ****p < 0.0001).

By lentiviral transfection, we constructed HeLa and SiHa cell lines overexpressing lnc-CNNM3-DT, and the overexpression level was verified by RT-qPCR. There were three groups, blank group, lnc-NC group, and lnc-CNNM3-DT overexpression group. CCK-8(**Figure 5**), wound healing (**Figure 6**), transwell invasion assays (**Figure 7A**), and flow cytometry (**Figure 7B**) indicated that there was no significant difference in cell proliferation, migration, invasion, and apoptosis between the blank group and lnc-NC group (ns, *p* > 0.05). Lnc-CNNM3-DT overexpression suppresses HeLa cells proliferation (Blank vs lnc-CNNM3-DT, *p*<0.0001; lnc-NC vs lnc-CNNM3-DT, *p*<0.001), migration (Blank vs lnc-CNNM3-DT, *p*<0.01; lnc-NC vs lnc-CNNM3-DT, *p*<0.001), and invasion (Blank vs lnc-CNNM3-DT, *p*<0.001; lnc-NC vs lnc-CNNM3-DT, *p*<0.0001), while enhancing apoptosis (Blank vs lnc-CNNM3-DT, *p*<0.05; lnc-NC vs lnc-CNNM3-DT, *p*<0.01). Lnc-CNNM3-DT overexpression suppresses SiHa cells proliferation (Blank and lnc-NC vs lnc-CNNM3-DT, *p*<0.0001), migration (Blank vs lnc-CNNM3-DT, *p*<0.001; lnc-NC vs lnc-CNNM3-DT, *p*<0.01), and invasion (Blank and lnc-NC vs lnc-CNNM3-DT, *p*<0.001), while enhancing apoptosis (Blank vs lnc-CNNM3-DT, *p*<0.01; lnc-NC vs lnc-CNNM3-DT, *p*<0.05).

### 3.5 CC cells overexpressing lnc-CNNM3-DT showed decreased intracellular copper levels and LIAS expression

**Figure 8:**
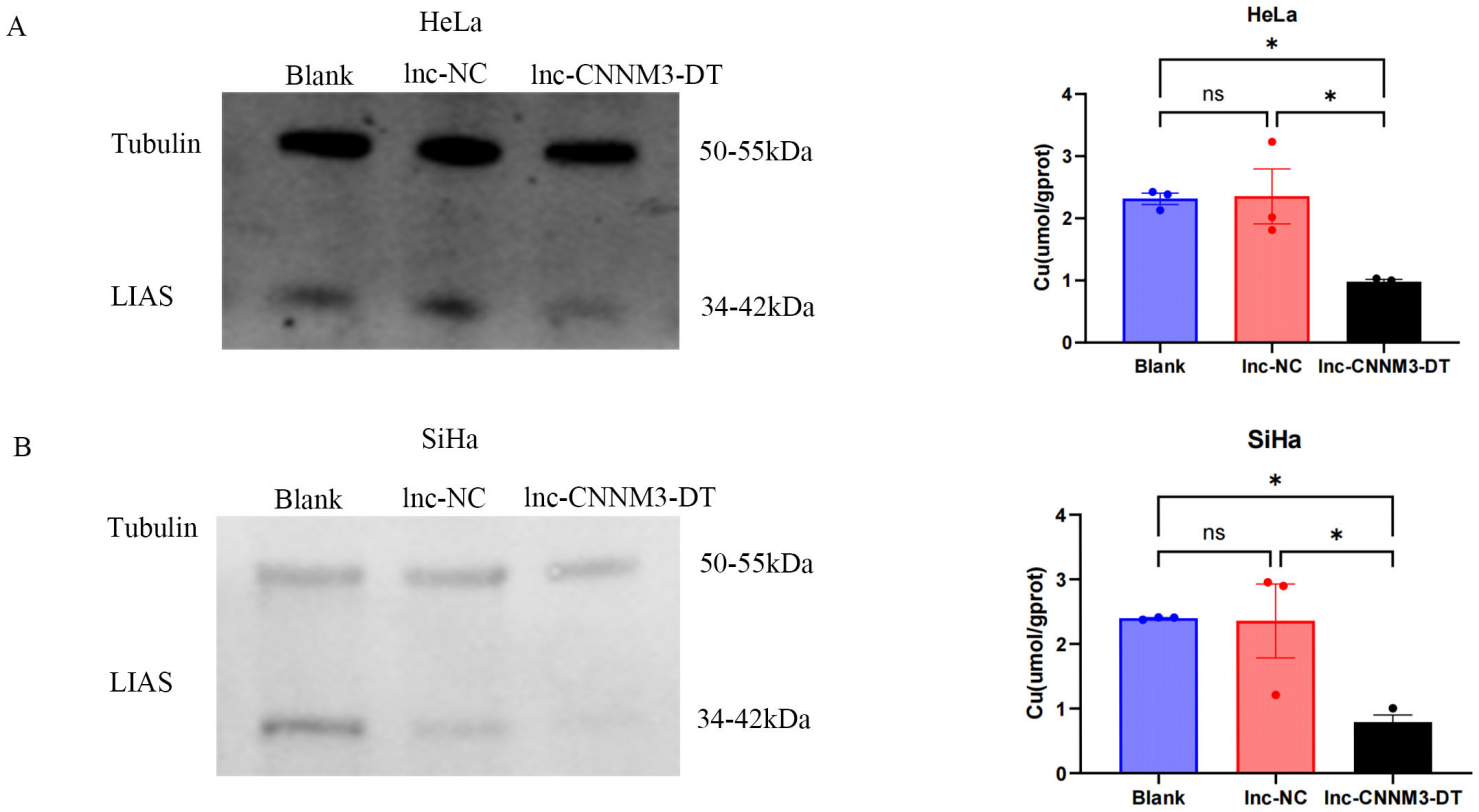
Expression of LIAS and intracellular copper ion content in HeLa and SiHa cells after overexpression of Inc-CNNM3-DT. Decreased copper ion content and LIAS expression in HeLa **(A)** and SiHa **(B)** overexpressing Inc-CNNM3-DT (ns, p > 0.05, *p<0.05).

As shown in **Figure 8**, HeLa and SiHa overexpressing lnc-CNNM3-DT showed decreased intracellular copper levels and decreased LIAS expression.Lnc-CNNM3-DT overexpression decreased intracellular copper levels in HeLa cells (Blank and lnc-NC vs lnc-CNNM3-DT, *p*<0.05). Lnc-CNNM3-DT overexpression decreased intracellular copper levels in SiHa cells (Blank and lnc-NC vs lnc-CNNM3-DT, *p*<0.05). The findings indicate that lnc-CNNM3-DT potentially suppresses cell proliferation, migration, and invasion while enhancing apoptosis by down-regulating LIAS expression and decreasing intracellular copper levels.

## 4 Discussion

Our research group previously downloaded RNA sequencing, clinical, and somatic mutation data of CC samples from TCGA database. CC patients were randomly assigned to training and testing groups. Prognostic models were determined in the training cohort using least absolute shrinkage and selection operator regression analysis and Cox regression models, and were validated in the testing cohort. Our study constructed a nomogram. Differences in biological functions were investigated through functional enrichment and immune function analysis. Tumor mutation burden (TMB) and tumor immune dysfunction and exclusion (TIDE) scores were used to predict responses to immunotherapy. Seven CRLs associated with CC prognosis were identified. Among them, five lncRNAs (AL360178.1, CDKN2B-AS1, ZNF667-AS1, AJ003147.1, and CNNM3-DT) were protective factors for CC patients, while two lncRNAs (ATP2C2-AS1 and BAIAP2-DT) were risk factors. Liu et al. (23) identified seven lncRNAs closely related to the prognosis of CC patients based on the TCGA database and Genotype-Tissue Expression Portal, constructing prognostic labels. The prognostic value of this label was validated using CC tissues. The seven CRLs: AL441992.1, LINC01305, AL354833.2, CNNM3-DT, and SCAT2 were protective factors, while AL354733.3 and AC009902.2 were risk factors. In conjunction with Liu’s findings, lnc-CNNM3-DT was selected for subsequent experiments.

These results suggest that lnc-CNNM3-DT acts as a protective factor for CC. This aligns with both our group’s prior bioinformatics analysis and the findings of Liu et al. (15). LncRNA is important in the normal development of organisms and tumorigenesis. During tumor development, lncRNA can act as tumor suppressor genes or oncogenes, leading to up-regulation or down-regulation of specific lncRNA relative to normal tissues (24). Some short open reading frames of lncRNA can encode neuropeptides that modify N6-methyladenosine, tumor angiogenesis, tumor metabolism, and signaling, with potential clinical value (25). In addition, lncRNA interacts with microRNAs (miRNA/miRs), mRNAs, proteins, and genomic DNA to produce physiological or pathological effects (24, 26). These regulatory RNAs are potential targets for cancer therapy because of their tissue and tumor specificity. Dysregulation of multiple lncRNAs has been associated with different types of cancer, including CC, breast, ovarian, and prostate cancers. Aberrant expression of multiple lncRNA has been found in CC, such as HOTAIR (27), H19 (28), GAS5 (29), CCAT2 (30), ANRIL (31), lncRNA LET (32), and lncRNA-CCHEL (33). Some lncRNAs (such as PTENP1, MEG3, and ZNF667-AS1) were downregulated in cancer samples. LncRNAs are also the regulatory molecules in cancer-related pathways such as Hedgehog, Wnt, Notch, PI3K/AKT/mTOR. It can regulate the plasticity of cancer stem cells as well (24). This suggests that lncRNA can be used as biomarkers to detect tumors, monitor prognosis and therapeutic targets for cancer management. Like Liu et al., Yu et al. (34)showed that lnc-CNNM3-DT was highly expressed in normal bladder epithelial cells compared with bladder cancer cell lines through bioinformatics analysis and RT-qPCR experiments. These results suggest that lnc-CNNM3-DT acts as a protective factor in tumors. FDX1 and LIAS are both key proteins in the regulation of cupropsis. Quan et al. (35) found that FDX1 expression was down-regulated in liver cancer tissues, LINC02362 combined with miR-18a-5p and directly regulated its expression, and FDX1 was the target of miR-18a-5p. We speculated that lnc-CNNM3-DT also regulates the CC process through a similar pathway. At present, lnc-CNNM3-DT could not query more information, so the potential miRNA could not be found for further research.

Research on bioinformatics databases indicates a correlation between LIAS expression and immune cell infiltration. In mice with pulmonary fibrosis overexpressing LIAS, inhibition of NF-kB attenuated chronic inflammatory responses, as indicated by increased Treg cell numbers and decreased T-cell infiltration (36). Similarly, increased Treg numbers and decreased T-cell infiltration were found in a mouse model of atherosclerosis overexpressing LIAS (37). In the tumor microenvironment, elevated Treg cells suppress effector T cell activation and function, facilitating tumor cell immune escape (17, 38).LIAS significantly influences tumor growth, blood vessel formation, and immune evasion (15).The overexpression of Lnc-CNNM3-DT significantly reduces the expression of LIAS. In lung adenocarcinoma, comprehensive analysis revealed that other copper death-related genes, including LIAS, are associated with immune infiltration and prognosis; their expression can affect the tumor microenvironment, influence immune cell infiltration, and potentially impact the response to immunotherapy (17). Furthermore, pan-cancer analysis of copper death-related genes indicated that LIAS mutations are associated with poorer survival outcomes in certain cancers, such as breast cancer (39). Therefore, lnc-CNNM3-DT may further inhibit the metabolic activity and antioxidant capacity of CC cells by downregulating LIAS expression, thereby enhancing its anti-cancer effects.

Many studies have found that various malignant tumors have abnormal accumulation of Cu, and the serum copper levelsof patients with drug-resistant tumors is 130% to 160% higher than that of patients with sensitive tumors (40). Cu significantly contributes to tumor cell proliferation, metastasis, and angiogenesis (41, 42). In addition, many studies have shown that CC tumor tissue has a higher copper levelsthan normal tissue (43).In our study, it was found that after overexpression of lnc-CNNM3-DT, intracellular copper levels, and LIAS expression were decreased, cell proliferation, migration, and migration ability were inhibited, and apoptosis was increased. The lnc-CNNM3-DT associated with cupropsis may decrease cellular copper levels and inhibit tumor cell growth by down-regulating LIAS expression.

## 5 Conclusion

We found and verified that the CRL CNNM3-DT may be negatively correlated with the progression of CC. It may inhibit the progression of CC by down-regulating the expression of LIAS and reducing the intracellular copper levels. Lnc-CNNM3-DT is a protective factor for CC and may influence the immune microenvironment in CC, potentially serving as a therapeutic target and a reliable biomarker for predicting the efficacy of immunotherapy in CC patients. The limitation of this study is that there are no reply experiments and animal experiments to prove the completeness and reliability of this hypothesis. Only 24 clinical samples were collected, which is not large enough to represent the real situation of most patients. The potential pathway study could not be performed because no lnc-CNNM3-DT interacting miRNA could be found by consulting existing databases. Future research will include recycling and animal experiments for further validation.

## Data Availability

The datasets presented in this study can be found in online repositories. The names of the repository/repositories and accession number(s) can be found in the article/supplementary material.
